# Treatment rates and delays for mental and substance use disorders: results from the Australian National Survey of Mental Health and Wellbeing

**DOI:** 10.1017/S2045796025000034

**Published:** 2025-02-14

**Authors:** Louise Birrell, Katrina Prior, Joshua Vescovi, Matthew Sunderland, Tim Slade, Cath Chapman

**Affiliations:** Matilda Centre for Research in Mental Health and Substance Use, The University of Sydney, Sydney, NSW, Australia

**Keywords:** anxiety, depression, mental health, substance use, treatment delay, treatment seeking

## Abstract

**Aims:**

Prompt initial contact with a treatment provider is a critical first step in seeking help for a mental or substance use disorders (SUDs). The aim of the current study was to provide estimates of patterns and predictors of delay in making initial treatment contact based on the recently completed Australian National Survey of Mental Health and Wellbeing.

**Methods:**

Data came a nationally representative epidemiological survey of *n* = 15,893 Australians. Measures included DSM-IV lifetime diagnoses of mood (MD), anxiety (AD) and SUDs; age of disorder onset; and age of first treatment contact. Correlates of treatment delay were examined.

**Results:**

SUDs exhibited the lowest lifetime treatment rate (27%), compared to MD (94%) and ADs (85%). Individuals with AD experienced the longest delay in seeking treatment (Mdn = 11 years), followed by those with SUDs (Mdn = 8 years) and MDs (Mdn = 3 years). Females had higher odds of seeking treatment for MD and AD but lower odds for SUDs. Recent birth cohorts showed increased treatment seeking across disorders, and higher education was associated with increased treatment seeking for MD and AD. Age of onset, country of birth and co-occurring disorders had mixed associations with treatment seeking.

**Conclusions:**

The study reveals stark disparities in treatment-seeking behaviour and delays across mental and substance use disorders, with a pronounced underutilization of services for SUDs. Additionally, attention should be directed towards early intervention for individuals with earlier symptom onset, those from earlier cohorts and those with co-occurring SUDs.

Mental and substance use disorders (SUD) are highly prevalent and cause profound suffering, with substantial societal and economic costs (GBD 2019 Mental Disorders Collaborators, 2022; Slade *et al.*, 2024; Whiteford *et al.*, 2013). While most people eventually receive treatment (approximately 80%, depending on the disorder) (Wang *et al.*, 2005), it is rarely sought or provided in a *timely* manner (Vaingankar *et al.*, 2013; Wang *et al.*, 2007). Global estimates from the World Health Organization World Mental Health Survey Initiative show individuals usually make first treatment contact many years after initial onset of a mental or SUD (Borges and de Matos, 2007; Johnson and Coles, 2013; ten Have *et al.*, 2013; Wang *et al.*, 2007), although this varies widely across and within disorder classes. For those with an anxiety disorder (AD) who do eventually seek treatment, the median treatment delay ranges from 3 to 30 years, while those with a mood disorder (MD) wait between 1 and 14 years and those with a SUD have median delays between 6 and 18 years, with substantial variation across countries (Wang *et al.*, 2007). One reason for these variations is differences in age of onset between disorders (Solmi *et al.*, 2022), with an earlier onset associated with longer delay to first treatment contact (Bruffaerts *et al.*, 2007; Post *et al.*, 2010; Wang *et al.*, 2005).

Delays in seeking treatment are problematic for several reasons; they typically result in more entrenched disorders, and during this delay, single disorders can progress into multiple comorbidities (Olfson *et al.*, 2012). This can lead to disorders that are more clinically severe, complex and difficult to treat (Goi *et al.*, 2015). Several factors have been identified as associated with treatment delay, including male sex, higher education (Tan *et al.*, 2023) and being in an older cohort (Bruffaerts *et al.*, 2007; Wang *et al.*, 2005). To reduce treatment delay and improve early treatment rates, detection of factors associated with delay to treatment contact at the national level is critical.

Large community studies show people with SUDs, the most common being alcohol use disorder, have some of the lowest rates and longest delays to seeking care, representing considerable unmet need (Chapman *et al.*, 2015; ten Have *et al.*, 2013). There are several possible reasons why rates and delay are higher for SUDs, particularly alcohol use disorder (Venegas *et al.*, 2021) including (i) cultural normalisation of substance use (meaning problematic use may not be perceived as needing treatment by individuals), (ii) lack of access and high-costs for substance use treatment, (iii) a lack of awareness of effective treatments for SUD and (iv) stigma, both from others and self-stigma. Over and above the general stigma surrounding mental disorders, stigma towards people who use substances is widespread, including internalized stigma (Hammarlund *et al.*, 2018). In a large nationally representative US sample (the National Epidemiologic Survey on Alcohol and Related Conditions), the number one reason given by individuals with an alcohol use disorder who had thought about, but did not seek treatment, was the belief they “should be strong enough to handle it alone” (Cohen *et al.*, 2007). This belief about self-reliance is a particularly important target for public health efforts, as while some individuals with an alcohol use disorder will recover without treatment many do not (Bischof *et al.*, 2003; Wang *et al.*, 2005). Many economically developed countries, such as Australia, have invested in substantial public awareness campaigns aiming to decrease stigma and promote help-seeking for mental health conditions and SUDs over the past 10–20 years. However, up-to-date national estimates for treatment delays for common mental disorders are currently unknown and Australia was not included in the World Mental Health Survey estimates for treatment contact delay (Wang *et al.*, 2007).

Additionally, little is known about contemporary factors associated with treatment delay at the individual disorder level and whether these factors vary across type of mental or SUD. Such differences are important to help guide public prevention and early intervention campaigns, which could be tailored dependent on mental disorder type and predictor. The recent 2020–2022 National Study of Mental Health and Wellbeing (NSMHWB) affords an opportunity to explore treatment delays for common MDs, ADs and SUDs among a large contemporary national sample.

This paper aimed to

- Estimate the projected probability of treatment contact (within 1-, 5-, 10-, 15-, 20- and 50-years of disorder onset, and over their lifetime) for MDs, ADs and SUDs in the Australian population, as well as the median time to first treatment contact (i.e., duration of treatment delay) and,
- Identify factors associated with delay to first treatment contact for MDs, ADs and SUDs, including individual disorders within these classes.

## Method

### Sample

Data are from the 2020–2022 Australian NSMHWB; a nationwide epidemiological survey, detailed elsewhere (Slade *et al.*, 2024). Selection was based on stratified multistage clustered area probability sampling of households in urban and rural areas across all states and territories, excluding very remote areas and discrete Aboriginal and Torres Strait Islander communities. One householder aged 16–85 years was randomly chosen to be interviewed. A total of 15,893 participants were recruited (response rate = 52%).

### Measures

#### Treatment delay

Lifetime diagnoses of DSM-IV MDs (major depressive disorder, bipolar disorder, dysthymia and mania); ADs (agoraphobia with or without panic, panic, social anxiety, generalized anxiety [GAD], obsessive compulsive and post-traumatic stress disorder); and SUDs (alcohol use disorder, drug use disorder) were assessed using a modified version of the World Mental Health Composite International Diagnostic Interview (Kessler and Üstün, 2004). For each disorder, participants were asked the age they first experienced symptoms, defined as age of onset. Age of first treatment contact was determined by asking participants if they had ever consulted a medical doctor, or any other professional about symptoms of a given disorder, and if so, how old they were the first time they did so. Treatment delay was defined as the time (years) between age-of-onset and age of first treatment contact, estimated for all disorders listed above.

#### Covariates

Correlates of treatment delay included sex at birth (male, female), birth-cohort (age at interview, categorized in 10-year bands: 1992–2005;1982–1991;1972–1981; <1972), age of onset (categorized using quartiles: early; early-average; late-average; late (Chapman *et al.*, 2015; Wang *et al.*, 2007, 2005)), country of birth (Australia or other), education and presence of a co-occurring lifetime disorder from another diagnostic class. Education and presence of co-occurring disorder were considered as time-varying disorders and recorded for each individual person-year. Participants’ education reflects their current age, starting at 0–11 and increasing until their highest level of educational attainment is achieved: years 0–11; year 12; post-secondary (bachelor’s degree, advanced diploma and diploma, certificate); postgraduate (postgraduate degree and a graduate diploma or certificate). The time-varying presence of a co-occurring lifetime disorder from another diagnostic class (MD, AD, SUD), coded as 0 until year of onset, then coded 1.

### Statistical analysis

Unconditional discrete-time survival models generated the cumulative incidence curves separately for each disorder. Curves were used to estimate the probability of first treatment contact within 1-, 5-, 10-, 15-, 20- and 50-years from first onset, median number of years until first treatment contact from disorder onset and projected probability of lifetime contact for each disorder (if participants were followed >85 years).

The effects of socio-demographic and clinical characteristics on treatment delay were estimated using conditional discrete-time survival models for diagnostic class level only (e.g., any MD, any AD, any SUD). Models adjusted for age, sex, age-of-onset, education, country of birth and lifetime co-occurring disorders. Models estimated participants’ survival time as the number of years from onset of any individual disorder within a given diagnostic class to age at first treatment contact for any disorder within the same diagnostic class, or to age at interview, whichever came first. The unconditional models provided odds ratios (ORs) associated with treatment delay, a higher OR synonymous with shorter treatment delay. The individual contribution of each covariate was determined using Wald *F*-statistics and associated *p*-values. Standard errors and 95% CI for all analyses were obtained using the jackknife variance estimator for each model. All data analysis was performed in STATA (StataCorp, 2023).

## Results

### Probability of treatment contact and treatment delay

**Figure 1.**
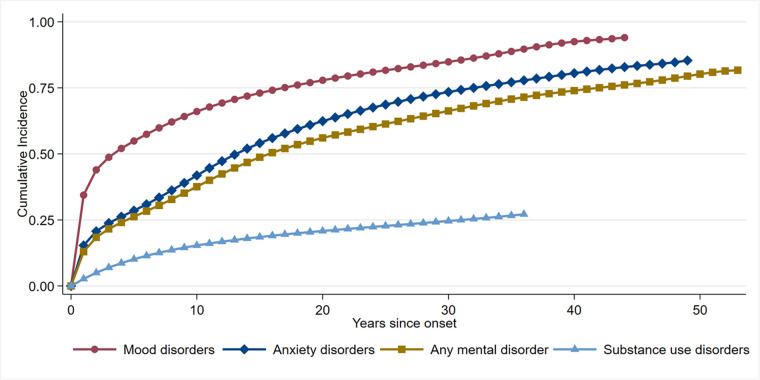
Cumulative incidence curves for time from symptom onset to time first sought treatment for each disorder class.

**Figure 2.**
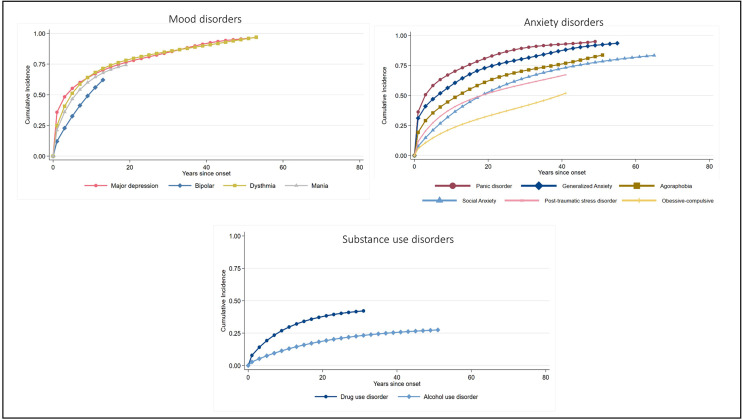
Cumulative incidence curves for time from symptom onset to time first sought treatment for individual disorders, within each disorder class.

Time to seek treatment curves for disorder classes are provided in Fig. 1, and for individual disorders in Fig. 2. Expected lifetime treatment rates and the proportion that made treatment contact at varying time since disorder onset are provided in Table 1.

**Table 1.** Projected probability of treatment contact since year of onset, and median duration of delay among those with lifetime DSM-IV mental disorders in the Australian population

	Projected probability of contact							Median delay
	Within 1 year	By 5 years	By 10 years	By 15 years	By 20 years	By 50 years	Lifetime	(years)^a^
*Any mood disorder*	0.34	0.55	0.66	0.73	0.78	–	0.94	3
Major depressive disorder	0.35	0.55	0.66	0.73	0.78	–	0.94	3
Dysthymia	0.24	0.54	0.65	0.72	0.78	–	0.84	3
Bipolar disorder	0.12	0.37	–	–	–	–	0.40	2
Mania	0.20	0.48	0.61	–	–	–	0.71	3
*Any anxiety disorder*	0.15	0.29	0.42	0.54	0.62	–	0.85	11
Generalized anxiety disorder	0.31	0.47	0.59	0.68	0.74	–	0.91	5
Panic disorder	0.38	0.57	0.69	0.77	0.82	–	0.88	2
Agoraphobia^b^	0.20	0.35	0.47	0.57	0.62	–	0.68	5
Social anxiety disorder	0.10	0.20	0.34	0.44	0.52	0.79	0.79	13
Obsessive compulsive disorder	0.07	0.15	0.21	0.29	0.35	–	0.41	10
Post-traumatic stress disorder	0.11	0.29	0.39	0.46	0.52	–	0.67	7
*Any substance use disorder*	0.03	0.10	0.15	0.19	0.21	–	0.27	8
Alcohol use disorder	0.02	0.08	0.13	0.16	0.19	–	0.25	10
Drug use disorder	0.06	0.21	0.30	0.35	–	–	0.36	4
*Any mental disorder*	0.13	0.26	0.38	0.49	0.56	0.80	0.82	12

a Median delay in years among those who eventually made treatment contact.

b Agoraphobia without panic disorder.

When looking at overall disorder classes, people with a SUD were the least likely to eventually seek treatment, with a lifetime estimate of 27%. In comparison, 85% of persons with any AD and 94% of persons with any MD were projected to make treatment contact across their lifetime. Among those who did seek treatment, the median time to seek treatment was longest for those with ADs (median delay = 11 years), followed by SUDs (median delay = 8 years), while those with MDs had the shortest delay in treatment seeking (median delay = 3 years).

There was wide variation across individual disorders in terms of the proportion who made treatment contact in the first year of onset; highest for panic disorder (37%) and lowest for alcohol use disorder (2%). The expected lifetime treatment rates also varied across individual disorders, from 25% for alcohol use disorder to 94% for major depressive disorder. Among those who eventually sought treatment, 50% of them did so within 2 years since symptom onset for panic disorder and bipolar disorder, whereas 50% sought treatment within 13 years since symptom onset for social AD.

### Factors associated with treatment contact

Table 2 shows factors associated with time to seek treatment. With respect to sex at birth, compared to males, females displayed significantly greater odds of seeking treatment and experiencing shorter treatment delays for any MD (OR = 1.38, 95% CI = 1.17–1.62) and any AD (OR = 1.47, 95% = 1.29–1.67) but had significantly lower odds of seeking treatment for any SUD (OR = 0.61, 95%CI = 0.49–0.75). For all disorder classes, people born in more recent birth cohorts (i.e., from 1973 onwards) had significantly higher odds of treatment seeking in comparison to people born prior to 1972 (ORs = 1.59–4.72). There was little evidence that education level was associated with treatment delay for SUD (ORs = 0.81–0.96) but was significantly associated with treatment delay for MDs (ORs = 1.45–1.69) and ADs (1.70–1.89), with higher odds of treatment seeking observed for those with a higher level of education.

**Table 2.** Factors associated with delay to first treatment contact among people with a disorder in the Australian population

	Likelihood of treatment contact								
	Any mood disorder			Any anxiety disorder			Any substance use disorder		
	OR	95% CI	*P*-value	OR	95% CI	*P*-value	OR	95% CI	*P*-value
Sex									
Male	[Ref]	[Ref]	[Ref]	[Ref]	[Ref]	[Ref]	[Ref]	[Ref]	[Ref]
Female	1.38	1.17-1.62	<.001*	1.47	1.29-1.67	<.001*	0.61	0.49-0.75	<.001*
Birth cohort									
<1972	[Ref]	[Ref]	[Ref]	[Ref]	[Ref]	[Ref]	[Ref]	[Ref]	[Ref]
1972–1981	1.59	1.24-2.02	<.001*	1.60	1.29-1.98	<.001*	1.72	1.22-2.40	.002*
1982–1991	1.81	1.45-2.26	<.001*	2.28	1.86-2.80	<.001*	2.51	1.86-3.40	<.001*
1992–2005	2.50	2.02-3.10	<.001*	3.89	3.16-4.78	<.001*	4.72	3.14-7.10	<.001*
Age of onset									
Early	[Ref]	[Ref]	[Ref]	[Ref]	[Ref]	[Ref]	[Ref]	[Ref]	[Ref]
Early-average	0.89	0.73-1.08	.226	1.09	0.95-1.25	.195	0.77	0.56-1.06	.111
Late-average	1.20	0.92-1.58	.177	1.24	1.04-1.47	.018*	1.04	0.76-1.43	.783
Late	2.27	1.63-3.17	<.001*	2.86	2.23-3.67	<.001*	1.55	1.11-2.16	.010*
Education									
Years 0–11	[Ref]	[Ref]	[Ref]	[Ref]	[Ref]	[Ref]	[Ref]	[Ref]	[Ref]
Year 12	1.45	1.14-1.84	.003*	1.70	1.40-2.05	<.001*	0.93	0.63-1.36	.685
Post-secondary	1.69	1.36-2.10	<.001*	1.63	1.39-1.90	<.001*	0.96	0.71-1.30	.775
Post-graduate	1.69	1.21-2.38	.003*	1.89	1.54-2.31	<.001*	0.81	0.51-1.31	.388
Comorbid mental disorder									
Any mood disorder	–	–	–	2.54	2.21-2.91	<.001*	2.09	1.62-2.70	<.001*
Any anxiety disorder	1.44	1.21-1.72	<.001*	–	–	–	2.08	1.59-2.71	<.001*
Any substance use disorder	1.21	1.02-1.43	.028	1.47	0.99-1.34	.077	–	–	–
Country of birth									
Australia	[Ref]	[Ref]	[Ref]	[Ref]	[Ref]	[Ref]	[Ref]	[Ref]	[Ref]
Other	0.80	0.69-0.93	.004*	0.74	0.64-0.85	<.001*	0.83	0.62-1.11	.198

* Significant compared to reference group at *P* < .05.

There were mixed results for the association between treatment seeking and age of onset, co-occurring disorders and country of birth. Those with a late age of onset, in comparison to an early age of onset, demonstrated significantly higher odds of treatment seeking for all disorder classes (ORs = 1.55–2.86), but there was little evidence of a difference for those with early-average or late-average onset compared to early onset (ORs = 0.77–1.20), except for ADs where those with late average onset had higher odds of treatment seeking compared early onset (OR = 1.24). The presence of any AD increased the odds of treatment seeking for those with any MD (OR = 1.44; 95% CI = 1.21–1.72) and vice versa (OR = 2.54; 95% CI = 2.21–2.91). Similarly, the presence of any AD or any MD increased the odds of treatment seeking for those with any SUD (ORs = 2.08 and 2.09, respectively). However, there was little evidence that the presence of SUD was associated with treatment seeking for any MD (OR = 1.21, 95% CI = 1.02–1.43) or any AD (OR = 1.47, 95% CI = 0.99–1.34).

## Conclusions

### Probability of treatment contact and treatment delay

Mental and SUDs are highly prevalent in Australia (Slade *et al.*, 2024) and worldwide (Scott, 2021). There is considerable variation in the probability of an individual making treatment contact dependent on the type of mental or SUD they experience. When considering rates of lifetime treatment contact, this study mirrors earlier findings from similar nationally representative surveys in general population samples using diagnostic interviews (Bruffaerts *et al.*, 2007; ten Have *et al.*, 2013) that the vast majority (94%) of people with any MD would seek treatment at some point during their lives. Specifically, the rate is the same as that found in other similar high-income countries, such as the US and Belgium, where approximately 9 out of every 10 people with an MD will eventually seek treatment (Bruffaerts *et al.*, 2007; Wang *et al.*, 2005). For those with any AD, 85% were estimated to eventually make treatment contact, although less than a third (27%) with any SUD would be expected to do so. For common individual MD and ADs, such as major depressive, GAD, social AD, post-traumatic stress and panic disorders, over two-thirds of respondents (67% to 94%) are likely to seek treatment in their lifetime, compared to only a quarter (25%) for alcohol use disorders. Comparing AD rates to those from other high-income countries that have used similar methodology, the current study found similar to higher overall treatment seeking rates to those in the Netherlands and Belgium where 61% and 85% of person’s with an AD are estimated to seek treatment in their lifetime, respectively (Bruffaerts *et al.*, 2007; ten Have *et al.*, 2013). There was substantial variation in lifetime treatment-seeking rates dependent on the individual AD experienced, ranging from a maximum of 9 out every 10 people with GAD eventually seeking treatment, to only 4 out of every 10 people with obsessive compulsive disorder seeking treatment over their lifetime. This variation across ADs is commonly observed across the globe, with estimates in the current study similar to the US population in which people with GAD typically have the highest likelihood of seeking treatment over their lifetime (Wang *et al.*, 2005). Relatively low treatment-seeking rates for SUDs have also been observed in other countries (Wang *et al.*, 2007), but the estimate in the current study is higher than that observed in a nationally representative survey from Belgium in which only 17% of people with a SUD reported seeking treatment (ten Have *et al.*, 2013). These findings indicate a clear underutilization of services for SUDs and underscore the need for targeted interventions to bridge this treatment gap.

Our findings reveal substantial delays in seeking treatment for mental and SUDs across a national general population sample, with considerable variation depending on type and class of disorder. Among people who eventually made treatment contact, shortest delays to first treatment contact were typically observed for MDs, on average occurring within 3 years. In contrast, there were pervasive treatment delays for ADs and substance disorders, with the longest delays observed for ADs (averaging 11 years), particularly social AD (13 years). The delays in treatment seeking for SUDs (median 8 years) fell between those for ADs and MDs. These delays reflect unnecessary suffering for individuals, their families and society, and it is imperative governments and health systems adopts a proactive approach to addressing these long delays. When looking at the proportion of people who sought treatment within the first year of disorder onset, rates were highest for MDs (33%), followed by AD (15%) and SUDs (3%). Across individual disorders, treatment contact within the first year of onset was most common for panic disorder (37%) and major depressive disorder (35%), while it was notably low for alcohol use disorder, with only 2% seeking treatment within the first year. These patterns of timely (or untimely) treatment seeking reflect findings from previous studies (Bunting *et al.*, 2012; Chapman *et al.*, 2015; Stagnaro *et al.*, 2019; Vaingankar *et al.*, 2013; Wang *et al.*, 2007), particularly with regards to low rates of contact within the first year of onset for alcohol use disorders.

The estimated timeframes for seeking treatment for MDs are relatively short and largely consistent with comparable studies conducted in Europe, the USA, Asia and New Zealand (Bruffaerts *et al.*, 2007; Bunting *et al.*, 2012; Wang *et al.*, 2007). However, time-to-treatment for any AD was shorter in Australia compared to most other countries (which range from 10 to 30 years), except for Israel (3 years) (Bruffaerts *et al.*, 2007; Bunting *et al.*, 2012; França *et al.*, 2023; Wang *et al.*, 2007). Secondly, estimates of the median treatment delay for alcohol use disorders in the current study are notably shorter than previous Australian estimates from data collected in 2007 (10 years vs 18 years) (Chapman *et al.*, 2015) but are comparable to other countries (Borges *et al.*, 2007; França *et al.*, 2023; Tan *et al.*, 2023; ten Have *et al.*, 2013; Wang *et al.*, 2005). The fact that treatment delay is shorter now compared to 15 years ago may indicate increased awareness and education around the impact of alcohol use, particularly in more recent cohorts of young people, leading to earlier recognition of symptoms and treatment initiation. It could also reflect changes in cultural norms around heavy drinking, evident in a slow but continued trend of gradually declining risky drinking in Australia since 2004 (Australian Institute of Health and Welfare, 2024). However, while estimates of overall prevalence of alcohol use disorders are lower in the current study than those observed in 2007, severity among those who meet criteria is higher (Slade *et al.*, 2024). The fact that among a group with increased severity, rates of lifetime treatment-seeking are low (25%; which is considerably lower than 2007 rates of 34.6% (Chapman *et al.*, 2015)) and delays, although shorter, remain substantial (10 years), highlights the continued importance of reducing barriers to care for those with alcohol use disorders, including low perceived need or low problem awareness (Grigg *et al.*, 2023), difficulty accessing services (Dunlop *et al.*, 2020) and stigma (Dannatt *et al.*, 2021).

### Factors associated with first treatment contact

Similar to extant literature (ten Have *et al.*, 2013; Vaingankar *et al.*, 2013; Wang *et al.*, 2005), sex differences in treatment-seeking patterns were notable, with females demonstrating a higher likelihood of seeking treatment and experiencing shorter delays for MD and ADs. Conversely, for SUDs, females were less likely to seek treatment and experienced longer delays, compared to males. These disparities may be influenced by stigma surrounding mental health and substance use, which is particularly pronounced in women (Meyers *et al.*, 2021). For instance, two US studies found women with an alcohol use disorder were more likely to report perceived stigma as a barrier to treatment (Khan *et al.*, 2013; Verissimo and Grella, 2017).

Encouragingly, younger cohorts showed higher odds of seeking treatment and shorter delays. For example, those born between 1992 and 2005 were over four times more likely to seek treatment for a SUD, over two times more likely for an MD and nearly four times as likely for an AD, compared to those born before 1972. This positive trend suggests current generations of young people are more inclined to seek timely treatment than their parents’ generation; a finding replicated worldwide (Bruffaerts *et al.*, 2007; Wang *et al.*, 2007). It could be speculated that increased education, often embedded through schools, in conjunction with national mental health literacy campaigns targeted at young people, as well as more easily accessible online information, could also be contributors to this cohort effect. Such factors may empower young individuals to recognize symptoms early and seek help more promptly. In addition, Australia has significantly invested in the design and scale up on youth mental health services for those aged 12–25 years in the form of community hubs, although future evaluation research is needed to assess its potential impact on youth treatment seeking.

Age of onset emerged as a significant predictor of first treatment contact, with late onset being associated with higher odds of seeking treatment and shorter delays across all disorder classes, a pattern consistent with established literature (Bruffaerts *et al.*, 2007; França *et al.*, 2023; Tan *et al.*, 2023; ten Have *et al.*, 2013; Wang *et al.*, 2007). One plausible explanation for this is that disorders which have an earlier age of onset, such as ADs (Solmi *et al.*, ), may not be easily identifiable by younger individuals and thereby rely on parental recognition and proactive facilitation of help-seeking behaviours. Equipping parents with knowledge and resources to better recognize mental health issues in children could mitigate key barriers to initiating treatment, particularly for early-onset disorders (Reardon *et al.*, 2017). Higher education level was also associated with treatment-seeking for MDs and ADs but not for SUDs. Co-occurrence of disorders also influenced treatment-seeking likelihood and delay, with the presence of an MD or AD increasing the odds and decreasing the delay of seeking treatment for the other two disorder classes. This suggests that co-occurring MDs and ADs might serve as indicators of severity and are drivers of treatment among those affected by multiple disorders. This aligns with prior research that indicates the greater the number of MD and ADs experienced alongside a SUD, the more severe and debilitating the symptoms can be, and the higher the rates of treatment seeking/utilization (Prior *et al.*, 2016). In contrast, the presence of a SUD was not associated with treatment seeking for MDs or ADs.

### Limitations

This is the first and most comprehensive Australian epidemiological study to examine probability of treatment contact, delay to seeking treatment, as well as factors associated with the time-to-seek help for individual mental and SUDs. However, we note several limitations. Firstly, the cross-sectional survey relied on retrospective recall of disorder onset and first treatment contact. This introduces the possibility of recall bias, as respondents may have failed to recall events occurring over their lifetime or dated them inaccurately but is in line with other studies of this nature (Wang *et al.*, 2007). There were also small sample sizes for some individual disorders, introducing a degree of uncertainty into estimates. As per previous studies (Olfson *et al.*, 2012; Tan *et al.*, 2023; ten Have *et al.*, 2013; Vaingankar *et al.*, 2013; Wang *et al.*, 2007), the current study focuses on treatment seeking and first treatment contact, without ascertaining whether treatment was actually received and, if so, about the type, intensity, or effectiveness of these treatments. Additionally, the study was unable to consider a variety of other factors that may affect treatment contact, such as time-varying factors of household income, socioeconomic status and employment status, thereby limiting the depth of our understanding of treatment-seeking behaviour and its determinants.

Finally, given the timing of data collection (2020–2022), the potential impact of the COVID-19 pandemic is noted. Several scoping and large systematic reviews of the impact of COVID-19 on mental health in Australia and globally have found increases in the prevalence of mental health problems (particularly anxiety and depression) and poorer wellbeing during the pandemic (Blendermann *et al.*, 2024; Bower *et al.*, 2023; Xiong *et al.*, 2020; Zhao *et al.*, 2022). Some groups (e.g., young people, women and financially disadvantaged) were disproportionally affected. While the precise impact of COVID-19 on treatment-seeking rates and unmet need for treatment remains uncertain, it is unlikely to have had a huge impact when considering lifetime treatment-seeking rates.

### Conclusion

While most individuals with mental disorders eventually seek treatment, this varies considerably across individual disorders, and the delays from initial disorder onset to first seeking treatment can be extensive. Despite being a high-income country with considerable resources, Australians are waiting an average of 12 years before seeking treatment for a mental or SUD. This highlights the need for continued investment in understanding and addressing the barriers to seeking timely care, as well as a focus on prevention and early intervention, including early screening for common disorders.

## Data Availability

The detailed microdata used in this study can be obtained by seeking approval from the Australian Bureau of Statistics.
